# ‘Better she gets infected by other diseases but not pregnancy’. Narratives from adolescent girls and community males following pregnancy escalation during COVID-19 lockdown

**DOI:** 10.3389/frph.2025.1643865

**Published:** 2025-08-21

**Authors:** Linda Mason, Enid Awiti, Sophie Young, Fredrick Otieno, Garazi Zulaika, Penelope Phillips-Howard, Supriya D. Mehta

**Affiliations:** ^1^ Department of Clinical Sciences, Liverpool School of Tropical Medicine, Liverpool, United Kingdom; ^2^ Nyanza Reproductive Health Society, Kisumu, Kenya; ^3^ Division of Epidemiology & Biostatistics, School of Public Health, University of Illinois Chicago, Chicago, IL, United States; ^4^ Department of Internal Medicine, Division of Infectious Diseases, Rush Medical College, Chicago, IL, United States

**Keywords:** qualitataive analysis, pregnancy, abortion, AGYW, Kenya, family planning (FP), males and females

## Abstract

**Introduction:**

Adolescent sexual and reproductive health in low- and middle-income countries is critical to address following the COVID-19 pandemic. Growing evidence of its’ impact includes increased teenage pregnancies and higher rates of unsafe abortion. Our qualitative study sought to understand perspectives and behaviours around these escalations.

**Methods:**

Using random sampling we conducted focus group discussions with adolescent girls and young women (AGYW) from western Kenya to understand perceptions and behaviours that drove high rates of pregnancy and abortion. Alongside, male opinions were sought using opportunistic sampling to recruit participants.

**Results:**

Six FGDs with AGYW (*n* = 54) and five with community males (*n* = 53) were conducted with data analysed using thematic analysis. Results were grouped under 4 themes: (1) Fear, responsibility and blame; (2) Drivers of abortion; (3) Accessing an abortion; (4) Family planning including condom use. AGYW narratives revealed a dread of unintended pregnancy fearing parental and community reactions whilst men feared unfair blame from the community for impregnating AGYW despite admissions of sexual relationships with schoolgirls. Abortion attempts were common, girls described clandestine bids, including ingestion of dangerous or ineffective products, methods corroborated by the men. Many participants, male and female did nothing to mitigate pregnancy risks, disliking condoms and perceiving family planning as a threat to future fertility, or giving license to girls’ promiscuity.

**Conclusion:**

AGYW remain at high pregnancy and unsafe abortion risk until community attitudes and knowledge are challenged. Our findings highlight the need for information and education to dispel myths and misinformation regarding family planning methods, and address inequities in gender norms. Safe, legal and affordable abortion is also paramount. Follow-up is required to assess long term physical and psychological consequences of the high number of unwanted pregnancies and abortions, particularly amongst those who had a failed abortion.

## Introduction

Adolescent girls and young women (AGYW) from low- and middle-income countries (LMIC) are vulnerable to poor sexual and reproductive health (SRH), with potential impact across the life course including adverse effects on their offspring. Nearly one third of adolescent girls in LMIC become mothers by the age of 19 (1). Complications arising from pregnancy, childbirth and unsafe abortion are among the leading causes of mortality among AGYW aged 15–19 (2, 3). As of 2019, an estimated 21 million pregnancies occur annually to girls aged 15–19 in LMIC: approximately half are unintended, while one quarter of these pregnancies end in, mostly unsafe, abortion (4). Both young age and unintended pregnancy are associated with long-term social and economic harms, including disproportionately high rates of school-drop out, child marriage, social isolation and reduced life-time earnings. Adolescent pregnancies are associated with lower use of antenatal services, and greater risk of postpartum depression (5). Of great concern is having an unsafe abortion, with AGYW more likely to delay the procedure, resulting in termination of pregnancy after their first trimester when health risks are far greater (6). Adolescents are also more likely to seek abortion from unlicensed providers or attempt to induce an abortion themselves (2, 7). For adolescents continuing with their pregnancy, aside from the long term marital, social and economic consequences, risks to young mothers include higher rates of eclampsia, sepsis and obstetric fistula, while babies born to them are more likely to be preterm and low birth weight (8).

Designated as high priority before the COVID-19 pandemic (9), adolescent SRH in LMIC has become even more critical to address post pandemic. Lockdowns and containment measures resulted in the closure, restriction, or relocation of SRH services including safe abortion clinics (10), and disruption to the supply chain of hormonal contraceptives and condoms. Moreover, increased poverty and economic instability intensified the SRH crisis (11). School closures curtailed Comprehensive Sexuality Education (CSE) programs **(**12). Evidence of the damaging impact of the pandemic on adolescent SRH in LMIC is mounting, with reports of increased teenage pregnancies alongside higher rates of unsafe abortion and child marriage, the latter projected to increase by 13 million over the next decade (13).

In Kenya, where the present study is set, the few studies undertaken to date evidenced a spike in number of pregnancies and other detrimental SRH outcomes since COVID related closures. Limited access to youth friendly or SRH clinics (14), and difficulty accessing contraception increased during this time (15). As a result, pregnancy amongst AGYW in Nairobi more than doubled during 2020 compared with pre-pandemic levels (16) In rural western Kenya, Zulaika et al. (17) reported that schoolgirls experiencing lockdown measures had twice the risk of falling pregnant in comparison to the prior school cohort and were also less likely to report their first sex as desired. In a subset of the same western Kenya secondary schoolgirl cohort, Mehta et al. (18) reported a 36% increase in relative prevalence of STIs post pandemic. Congo et al. (19) reported a 60% increased pregnancy risk to AGYW during lockdown with a quarter ending in abortion in their 3-site study in Thika, Nairobi and Kisumu.

Our prior qualitative paper in western Kenya (20) reported on the AGYW and community males’ perceptions that sexual activity and number of partners increased during COVID restrictions as acute poverty drove transactional sex, whilst increased leisure time added opportunity. Consequently, pregnancy rates were believed to escalate. *The objective of this study was to investigate the perceptions of AGYW and community men in response to these escalating pregnancy rates. We included opinions of men from the same communities acknowledging their dual role as main wage earners and heads of household in this patriarchal society, and as (potential) sexual partners of AGYW in the community. Specifically, we wanted to explore recent experience and perceptions of pregnancy, abortion and family planning. It is intended that this information may be utilised in future epidemics or similar circumstances to prevent high rates of pregnancy occurring in AGYW*.

## Materials and methods

This paper utilizes data from the focus group discussions (FGDs) reported previously (20) using a deductive approach to focus on the data pertaining to pregnancy, including abortion and family planning. Briefly, the study took place with residents of Siaya County, western Kenya. With an estimated population of 153,000, the predominantly Luo population comprises subsistence farmers, fisherfolk, and gold miners (21).

### Ethical consideration

This study was approved by the ethical review boards of Rush University Medical Center (22011311), Maseno University Ethical Review Committee (MSU/DRPI/MUERC/01021/21), and University of Illinois Chicago (2022-0220). Liverpool School of Tropical Medicine (21-087) reviewed the ethical approvals and gave favourable opinion. All participants gave written consent to participate in the study.

### Recruitment

The AGYW were participants aged 14–25 years at enrollment in the Cups and Community Health (CaCHe) study, a sub-study of the Cups or Cash for Girls (CCG) Trial (22). CaCHe evaluated the effect of menstrual cups on the vaginal microbiome, Bacterial Vaginosis (BV), and STIs (21). In 2018, 436 girls were enrolled, with survey and specimen collection visits every 6 months. In 2022, at the 48-month visit, field staff asked AGYW if they wished to participate in a FGD study. Among those expressing interest, stratified simple random sampling was utilized to invite potential participants and ensure representation across the study area. For recruitment of men living in the same areas as the AGYW, we targeted motorcycle taxi drivers (boda boda), fisherfolk, miners, and farmers as being representative of the primary employment sectors in the local community. Opportunistic sampling was used with additional snowball sampling to enhance community men's group sizes as needed. The moderator contacted each participant by telephone to invite them to take part providing an outline of the study and what participation would involve. She also answered any questions they had but had no additional relationship with them. Aiming for 8–10 participants per FGD, we invited 12 to each session allowing for non-attendance. Participants who did not attend were not followed up to find out reasons for this. Our original aim was to conduct ∼8 FGDs, 4 of each gender. However, two further FGDs with AGYW and one with men were conducted to ensure saturation was reached, with no new themes emerging and all conceptual categories in the guides covered.

### Focus group discussion approach

Two semi-structured FGD guides were developed in tandem, with overlapping topics facilitating comparison between AGYW and men. The guides focused on participants’ perceptions and behaviours pertaining to sexual relationships and SRH including the period following COVID-19 related school closures and onwards. Specific questions explored high rates of pregnancy and community response, as well as consequences and subsequent behaviours, including pregnancy, abortion and contraception use. Prompts were added where relevant, and further questions included in light of new findings emerging during the course of the FGDs.

### Procedure

FGDs began with discussion of the study purpose, after which participants provided written informed consent in their language of choice (English, Swahili, or Dholuo), which included consent for audio recording of the discussion. Each participant was assigned a number instead of name to protect confidentiality (denoted in the quotes, e.g., Girl 3). The discussion was carried out in the participants’ preferred language. The FGDs were transcribed verbatim and any Dholuo or Swahili was translated into English. A female Kenyan moderator (EA) with prior experience in conducting FGDs led each discussion, with a male notetaker used for discussions with men, and a female notetaker for FGDs with AGYW. The moderator is also knowledgeable about the socio-cultural context of the study setting and community. The FGDs were conducted in a central location (e.g., community hall or church) and lasted 1.5–2 hours. Some of the AGYW brought their children, but no other members of the public were present.

### Analysis

The transcripts were originally reviewed and compared against the original audio for accuracy by the moderator. Transcripts were analyzed using NVIVO version 12 Pro Software. Then, because we wanted to develop nuanced understanding of specific themes that arose from our previous analysis, we adopted a deductive approach. This involved mining the data *a priori* for content pertaining to pregnancy, abortion and family planning. From then, a thematic analysis ensued (23). Any relevant content from our transcripts that fit under one or more of these headings were coded. Codes were then grouped together where relevant and if applicable, the codes were reassigned with a new name. When all relevant data were coded, the refined groups of codes were assigned to one or more of the four themes we identified. A narrative framework was written around the codes/themes to reflect the emergent findings, with further analysis including comparing and contrasting participant views both within and across the genders and the individual FGDs. Repetition of opinion and experiences, language used – expression and tone, group dynamics were also noted. As the narrative progressed, key quotes were added to illustrate the points made. A reflexive process completed this analysis whereby the raw transcripts were revisited to ensure key quotes were not taken out of context, that the narrative comprised a fair representation of participant views, and that no salient points had been missed. Team members who were familiar with the transcripts (EA and SY) read the resulting narrative as a quality check suggesting minor amendments where appropriate.

## Results

Eleven FGDs were conducted with 107 participants: six with AGYW (*n* = 54) and five with community males (*n* = 53). All participants were of Luo ethnicity with the exception of one Luhya male. (Participants’ characteristics are summarized in Table 1. Distribution of FGD participant characteristics).

**Table 1:** Distribution of FGD participant characteristics.

Focus group characteristics	AGYW, *N* = 54		Community Men, *N* = 53	
	Number of participants	Duration	Number of participants	Duration
FGD				
FGD 1	11	2 h, 11 min	11	2 h, 15 min
FGD 2	8	2 h, 17 min	12	2 h, 15 min
FGD 3	11	2 h, 50 min	10	2 h, 38 min
FGD 4	10	2 h, 0 min	12	2 h, 0 min
FGD 5	8	2 h, 10 min	8	2 h, 30 min
FGD 6	6	2 h, 22 min		
Participant Characteristics	*n* (%)		*n* (%)	
Mean age (range)	20.9 (18–24)		27.1 (19–41)	
Married	4 (7%)		25 (47%)	
Has children	13 (24%)		29 (55%)	
Currently in school	24 (44%)		1 (2%)	
Occupation (not mutually exclusive, some men had multiple jobs/occupations; AGYW had to report this as employment outside of home chores)	6 (11%)		53 (100%)	
	2 (4%) Farming		31 (58%) motorcycle taxi	
	1 (2%) Hotel work		13 (25%) Fisherman	
	1 (2%) Salon		8 (15%) Miner	
	2 (4%) Shop attendant		2 (4%) Farmer	
			3 (6%) Businessman	
			1 (2%) Artisan	
			4 (8%) Mechanic	

Results were grouped under 4 themes: (1) Fear, responsibility and blame; (2) Drivers of abortion; (3) Accessing an abortion; (4) Family planning including condom use.

### Fear, responsibility and blame

Girls’ narratives revealed unintended pregnancy to be a prevailing fear for them and their peers, whilst the men feared being blamed for their pregnancies. The anxiety for each group stemmed from the social ramifications and reactions from the community towards them.

The visibility of a pregnancy would display to everyone that a girl had been sexually active. Girls and men observed: ‘*With pregnancy you will be seen*’ (Girl 7 FGD 5); ‘*Pregnancy gives out evidence that you really did it’ (Girl 4 FGD 3); ‘Pregnancy is visible to everyone and you cannot hide’ (Male 8 FGD 2).* The feared consequences of pregnancy for the girls were harsh reactions from parents, including the possibility of being thrown out of the home, and humiliation and shame stemming from community judgement, including neighbours, elders or peers.

While acknowledging pregnancy could lead to school dropout, few participants spoke of the long-term consequences of a pregnancy. Considerations such as ‘*the baby will remain in your life forever*’(Girl 1 FGD 6) or ‘*at times there was no food, now if you get pregnant you would add more burden…you will be hungry together with the baby’ (Girl 5 FGD 6)* were rarely expressed, in contrast to the sentiments throughout the discussions of how the community condem the situation:

‘They only fear getting pregnant and HIV, only that can destroy their reputation in the village. You know you can have a disease, and you remain calm but with pregnancy it’s something that develops and everyone gets to observe it and some men will laugh at you’. (Girl X FGD 2)

‘There are two types of fear, one is from family because you are afraid of what your mother and father will say. Second is community, you know how people in the community are mouthy. So you sit down asking yourself “what will so and so say”, so you will have that fear’. (Girl 8 FGD 3)

When discussing the consequences of sex, girls’ narratives indicated that they were considerably more concerned about pregnancy than STIs and HIV, frequently using this analogy to demonstrate their anxieties.

‘They had more worries because the scariest thing for a lady is getting pregnant. In fact, better she gets infected by other diseases but not pregnancy’. (Girl 5 FGD6)

‘Nowadays it is being said that HIV is just a normal disease. They just take medicine and life continues. Now they only afraid of pregnancy but not HIV’. (Girl 2 FGD 4)

It was rare to hear a different opinion, the following atypical of a girls’ reaction:

I feel it is better to have a pregnancy instead of STI. According to me, in pregnancy you will carry it for 9 months and give birth and people will love the baby. And for the disease you will have it and will be affecting you. Sexually transmitted disease will eat you up, but pregnancy when you give birth you are done with it’. (Girl 8 FGD 3)

As well as noticing and commenting on the high rate of pregnancy and negative community views, the men were aware of potential consequences if they were held responsible for being the father to a schoolgirl pregnancy. However, they did not speak of concern for the girl and the consequences for her or her offspring.

As for us we also get a lot of money by the lake, so you can buy them anything they wish for. There are rules at the beach and mostly fishermen are a bit scared. It was decided that if you are found defiling a schoolgirl then you are liable for punishment by imprisonment for jail term not less than 20 years. This has made them fear the young school going girls’. (Male 8, FGD 2)

Men repeatedly complained that they were being blamed, voicing that their group of peers (i.e., as boda boda drivers, or fishermen for example) were unfairly held liable, and sometimes even apprehended for impregnating schoolgirls, but believed others such as girls’ schoolmates or teachers to be the culprits.

‘What you are saying that a schoolgirl when she gets pregnant or when she drops out of school it is a boda boda guy or the person who work in the lake/fishermen. It is not the truth. You can find that even in school she can be made pregnant by a teacher or even farmer it is not a must but it is a person working in the lake or boda boda guy’. (Male 9 FGD 2)

Nonetheless, men repeatedly spoke of their own and their peers’ sexual relations with young girls. One man also included the girls’ parents in his blame saying:

‘Even the builders could impregnate a child, not to mention teachers. But then I do blame the parents for their complacency because once their child has been defiled, they should take an immediate action. Instead, they would negotiate with the perpetrators because of greed for money. Personally, I have been in a relationship with a girl to an extent that I even shower the mother with gifts and money, but I later ended the relationship before late. So parents do not take the responsibility’. (M: How did you collude with the mother of the girl?) ‘I mean whenever she leaves her house she comes back with a packet of sugar. Suppose the father passed away and their financial situation is not that good. So I could take the girl out in the watch of the mother in the night, we could even exchange pleasantries with the mother before I leave with her daughter at that time of the night. This would continue until the girl gets pregnant thereafter the mother is unable to take any action against that’. (Male 11 FGD 4)

Girls corroborated that whilst boda boda operators and fishermen were responsible for many of the pregnancies, other actors included their male peers or older men, including sponsors. Girls’ statements about this were matter of fact attaching no sense of the blame that had emerged from the men's narratives.

Three of the men's FGDs noted that their professional organisations work to ensure members act appropriately and threaten legal action if a member was known to engage in sex with a minor. As one participant explained:

‘Among all the sectors, I would tell you that boda boda has the toughest laws of all. One, as for the stage where I operate, we have very stringent laws, one of them being you cannot speak to any girl in uniform. Two, since we know each other by names, if I find any of us having an affair with a schoolgirl it is my responsibility to disclose to the whole team so that he could face the recommended punishment. For example, in our stage we would take you to the police once we have confirmed that it is true that you are violating children’s rights. We would take you to the police or children’s department so that an action could be taken against you and this has made the boda boda not to be perpetrators in such cases’. (Male 12 FGD 4)

That work organisations consistently reacted to protect girls against defilement was contradicted in another discussion by a participant describing closing of rank to protect a fellow member of their organization. He stated:

‘It’s only that if it is a village mate as you asked in the group if he gets a girl pregnant, what we do is to cover it and say that the snake has entered the guard. For this one we can cover up ourselves and say that the snake has entered the guard so that I don't disclose someone there’s nothing like somebody has done something so it is a must cover up for ourselves so that this matter doesn't reach the elders or even the authorities so we have to cover so if we are here and we are all villagers’. (Male 10 FGD 1)

He then went on to explain that if the man was an outsider, then of course those in the organization would expose him. There was some mention by men, and corroborated by girls, that some men deny responsibility for a pregnancy, particularly if they are married, sometimes citing that the girl had other partners who might be responsible. Girls also acknowledged that in some cases they may not know who was responsible.

‘The issue about pregnancy brings a lot of complications more so when you have sponsored the lady for so long then unfortunately or fortunately, she becomes pregnant. Sometimes this happens when you are already married, she reports it and tells you that she is keeping the pregnancy because it is yours, she is going nowhere because it’s yours and she is going to stay with you. When she has told you such words and you have a family on the other side which doesn’t know anything between you and the lady, it is going to start stressing you out that if the lady gives birth, it is your responsibility, and this can result in chaos in your family. So that’s why people tend to neglect a lady when they become pregnant’. (Male 7 FGD 5)

### Drivers of abortion

Along with a perceived rise in pregnancy came the viewpoint that the number of abortions among AGYW also rose. Abortion was perceived as a very common outcome during this time. Some participants believed abortion was more common than continuing with a pregnancy.

‘There is a percentage that aborted because they became pregnant unexpectedly and sometimes, she got pregnant and doesn't want you to know about it. Most of the ladies I see get pregnant carrying to delivery is always a last option, and she has the ability to remove it, she can just abort it’. (Male 5 FGD 5)

In the discussions around abortion, the social ramifications of pregnancy again emerged prominently. Discussions indicated that it was the pregnancy itself that was shameful or embarrassing, rather than having the abortion, and ultimately the driver to seek an abortion.

Other reasons for having an abortion included pressure from the partner, or denial that he was responsible for the pregnancy, both acknowledged by AGYW and men. Similarly, both spoke of girls having multiple partners and consequently might not know who the father was. Young age and feeling incapable of motherhood, or just to be labelled as a mother was also cited by girls, as was wanting to continue their education. Rarely did girls mention the financial burden that continuing a pregnancy would bring, whilst men also did not appear to consider this’.

‘Some also looked at the background of the family, now maybe you don’t have much in your home, so they feel they should do abortion to avoid the high cost of life’. (Girl 1 FGD 6)

The following quotes highlight various reasons for abortion raised in the girls’ discussions, very little emerged on reasons for abortion in the male FGDs.

‘Then those who were aborting, some were doing that because they didn’t know who got them pregnant, sometimes the parents are strict and cruel, maybe if you go to report that you are pregnant you could be chased out of the house, you just decide to look for money to remove it or maybe the person who got you pregnant has told you to abort the pregnancy’. (Girl 3 FGD 5)

‘More [had an abortion than continued with pregnancy] because in the situation that the lady has many boyfriends and at the point of getting pregnant she couldn’t trace who is particularly responsible. And also, among the boyfriends none could also be willing to step up towards taking responsibility about the pregnancy. Most of them could deny being responsible. The situation would even get worse if the parents are very cruel, especially if she has to tell them that she is pregnant. This was something that really disturbed them’. (Girl 6 FGD 2).

‘Some people feel that they are still young, and they can’t be mothers… they feel that being called the mother of so and so does not sit well with them’. (Girl 5 FGD 6)

### Accessing an abortion

Participants, both AGWY and men indicated abortion was mainly accessed through private hospitals, either clandestinely via a doctor^1^, traditional healer, herbalist, or village elder or, cited more frequently, self-induced. Assistance from a pharmacist and a community health worker were mentioned in one instance each. While we did not comprehensively ascertain how girls selected the abortion approach, discussions highlighted cost, confidentiality, and effectiveness, with no clear pattern emerging. The price for abortion conducted through a hospital or doctor was mentioned as 3,000–5,000 Kenyan shillings (KSH; ∼$25–$38 USD). Herbalists/traditional healers were reported as somewhat lower cost, at approximately 2000 KSH (∼$15). A few noted that cost impacted choice of provider. Participants also mentioned that doctors or healers could set the price on an individual basis, suggesting this may take into consideration the length of gestation, but could be performed for free depending on negotiations.

‘What I know is that four thousand shillings for that thing, mostly for the doctors who do it backdoor’. (Girl X FGD 5).

Private hospitals were preferred over public hospitals as they were perceived to offer more confidentiality, and in some instances were nearer in proximity to AGYW homes. Parents, in particular mothers, were more likely to direct their daughters to private hospitals sometimes keeping this a secret from their husbands. One man reported ‘*Babas [fathers] are clean*’further explaining: ’*some men are very cruel, so the woman fears the husband coming to realize that their daughter is pregnant. That makes women contribute, and sometimes she is also against it but it forces her to take her daughter to be done for abortion so that the father never comes to think about it’.* (*Male 1 FGD 5).*

Girls described that they accessed doctors for abortion services secretly through their local networks, e.g., referral from a traditional healer. Sometimes girls who had previously sought abortion services directed their peers to them.

‘Most often, if you had a friend who had an abortion they would share with you their story, where and how they did it. After which if they did it in the hospital, they would take you to the doctor to assist you with that’. (Girl 3 FGD 2)

Generally, doctors’ services were not widely known ‘*it is done secretly because the girl may die then the doctor may get into problems*’ (Girl 4 FGD 6) hence ‘*I don’t know how they do it in hiding but they do*’. (Girl 4 FGD 4)

Herbalist and traditional healer services were often mentioned, although little detail was forthcoming about how they were accessed, how pregnancy was terminated, nor the success this method had. Conversely, many girls detailed personal and peer accounts of self-induced abortions. One girl admitted ‘*I took nine coartem tablets, I took tea leaves, I drunk concentrated juice but right now the baby is walking* (Girl 5 FGD 5*).* Girls detailed a variety of methods to induce a miscarriage, including ingesting: boiled water with concentrated tea leaves, ‘*quencher*’ [undiluted juice], ‘*Omo*’ [washing powder], ‘*Coartem*’ [Malaria treatment], ‘*Jik’* [bleach], Panadol [pain killer] or wines and spirits. These substances were mentioned multiple times, giving the impression they were frequently discussed or used. Men similarly spoke of these substances as abortifacients, although none spoke of assisting in an abortion attempt (nor did we probe on this). FGD conversations turned to failed abortions, with both girls and men frequently telling of cases where an attempted abortion became an emergency or lead to death.

‘She was just our age mate, form four during the time of Corona, so she went to visit her boyfriend, then she became pregnant. On coming back the parents became harsh, so she felt that the only way is abortion. She took traditional medicine in excess, so she bled and died’. (Girl 3 FGD 6)

‘Yes, there is one in Ndori here who tried to do abortion but it was late. The embryo had already formed, the attempt went wrong, only the legs came out and she had to be rushed to hospital before she died’. (Male 1 FGD 5)

### Family planning including condom use

Despite the pervasive anxiety of a pregnancy for girls, there were opposing views about using family planning methods: many girls and men were against it, although others felt it was useful. Two key reasons emerged in the argument against family planning. A number of participants, both AGWY and men, voiced that family planning use led to disinhibition, enabling girls to be free to have sex or to think of having sex.

‘It cannot help [education], when she is on family planning she will only think of having sex. When she is free she will have sex because she knows no one will know so issues of education will be little’. (Girl 8 FGD 3)

‘Family planning increases the urge to have sex and she could sleep with a man even twice a week. Those who are not under family planning could even stay for a month without sex. Family planning could lead someone to contracting diseases like STI or HIV/AIDS. Family planning is a bad thing and it should be abolished completely’. (Male 2 FGD 2)

Whilst a few participants spoke of various genuine side effects, such as heavy bleeding or headache, more commonly others, both girls and men, told of family planning methods likely causing sterility or future fertility problems. They perceived therefore that it should not be used until after a girl had given birth, or was married and had a family to ‘plan’ for. In this respect they distinguished family planning methods as different to methods of contraception such as the condom which was used to prevent an immediate pregnancy.

‘Family planning have their side effects as in it will reach a point or in life that you may want to have children and you were on family planning and it eats up the ova so when it reaches that point you are trying to conceive but you are unable to, so whom will you blame and yet it is you who destroyed your life’. (Male 3)

Those in favour of family planning (fewer in number) felt that preventing pregnancy was good, it reduced worries, facilitated education, or, as one girl mentioned, prevented heavy bleeding.

‘According to me I think it’s a good thing because it helps prevent cases of a girl getting pregnant and they can complete their studies and relieve their parents of the responsibility of taking care of their kids as they get back to school. And minimizes cases of school dropouts’. (Girl 3 FGD 2)

One man summed it up using two sides of the argument:

‘*Ok, everything that has an advantage must also have its disadvantage. One of the advantages of that thing, the embarrassment that would have come to the family that the daughter of so and so got pregnant, it has cleared. Then if the lady was also going to school, it has prevented her from being a laughingstock in the event that she got pregnant. But the disadvantage that I hear it has is that it spoils the uterus such that one after being married she can’t easily conceive and reproduce because of the effect of the drug on the, kindly help me those who went to school [laughter] uterus something like that*’. (Male 6 FGD 5).

From the narratives it was clear some girls used family planning methods, talking of ‘*being injected*’ (no other method was mentioned). Just one girls’ FGD and one male FGD mentioned the morning after pill, Postinor 2, known locally as ‘P2’. Some participants in the girls FGD disclosed they had never heard of P2 prior to the discussion.

When discussions turned to the use of condoms girls and men alike spoke negatively, many preferring not to use them. Frequently cited by both girls and men as causing stomachache in girls, other side effects such as allergy or itching were mentioned, as was preference for sex ‘*skin to skin*’. A couple of men stated ‘*I don’t know how to use them’*.

’Some do complain that if they use condoms it gives stomach ache or complications at the bottom of their belly’. (Girl 3 FGD 2)

‘Others say, once someone uses condom it is not sweet. Then there are those boys who if are your boyfriend may have that mentality of “I cannot use condoms, if I use it infects me, or maybe gives me blisters” eeh, things like that’. (Girl 4 FGD 2)

Of the few males who used or spoke positively of condom use, this seemed to be for their own protection. No mention was made of using condoms to prevent men from infecting girls with sexually transmitted infections.

‘First, we don’t know who these girls are. You cannot sleep with a girl without a condom. You should put on three at once because some of these girls have syphilis (Male 10 FGD 3)

‘I prefer we use condom. It is not good to have unprotected sex with someone other than your wife because they may be sick. So I prefer using It’. (Male 2 FGD 3)

## Discussion

This paper chronicles narratives from girls’ and men's discussions exploring AGYW and community males experience and perceptions of pregnancy, abortion and family planning. Findings highlight the acute fear girls have of falling pregnant, while at the same time maintaining negative attitudes towards family planning and condom use. We document the drivers of the perceived high numbers of abortions among AGYW during this time, along with methods adopted and subsequent outcomes. We also capture men's perceptions of their role and responsibility in relation to sexual activity and unintended pregnancies among AGYW.

One striking observation from the girls’ narratives were the repeated accounts of their fears of pregnancy. This appeared to be a predominant anxiety shadowing their daily lives, with two key aspects to this fear. Firstly, the fear generally was not related to a burden of early motherhood but primarily about how ‘others’ would vilify them for being pregnant, a situation also reported by Miller et al. (24). In Kenya traditional and religious values still prevail, including sex being for reproductive purposes only, premarital sex is frowned upon, with abstinence promoted in schools. These norms lead to a culture that maintains a silence around issues of sexuality and sexual behaviours (25). Additionally, Kenyan society is hierarchically structured, and patriarchy remains part of the social fabric. Village Chiefs and elders, still predominantly male (26), are respected and influential in public governance at grassroots level, including over domestic issues amongst villagers (27). Further, Kenyan society is collectivistic with the population highly interdependent on their social and extended family circles. Hence others’ views are seen as critically important. This sociocultural context likely explains why girls’ primary concern is others’ response to their pregnancy. Sustained efforts by those influential in Kenyan society, including policy makers, religious, health and education leaders, chiefs and elders need to drive immediate and positive change to educate and promote informed and safe sex. Without this, condemnation from others will remain a fear for girls, causing barriers to service access, early pregnancy, and unsafe abortion.

Despite the pervasive fear of pregnancy, AGYW voiced that family planning was undesirable. Their narratives mirror findings from our quantitative survey of the broader CaCHe cohort: girls reporting sexual activity, hormonal contraceptive use was just 7.6% at baseline, reaching 21.6% at 48 months. However, the majority (61%) of girls reporting hormonal contraceptive use had experienced a prior pregnancy. The most commonly reported method was implant (46% of hormonal contraceptive users), followed by 31% using the injectable, with the remainder reporting birth control pills (data not shown). The small body of literature describing high pregnancy rates during the COVID-19 pandemic in Kenya attribute this in part to restricted access to family planning and peripheral services as well as stock-outs of supplies (28). This was not raised in any of the narratives from our participants; instead, it appeared that SRH service restrictions during the pandemic had minimal effect because of low demand for contraception. In trying to unravel the incongruency that contraceptive use is uncommon, while the fear of pregnancy is immense, it seems that negative perceptions around contraception, such as causing infertility and suggesting sexual promiscuity were the strongest factors preventing use. The view that FP leads to behavioural disinhibition has been reported in in Hakansson et al's survey of teachers and peer-counsellors in Kenya (29). Family planning was not seen as suitable for girls but deemed appropriate only for those who were older, married and planning to have a family, or should only be used after a woman has already given birth, as it was perceived to cause infertility in a nulliparous girl. Although the Kenyan government officially supports CSE (30), abstinence is commonly taught (31) despite evidence that this approach is ineffective and risks short- and long-term harms to AGYW and their offspring (32). Lastly, both girls and men did not like using condoms, a common barrier to condom use globally (33, 34). Findings from our study, along with other literature reflect a continuing vital need to educate AGYW and men, along with the wider community, towards realistic and factual information about modern contraception allowing rational and considered decisions to be made by individuals and their partners.

There was relative lack of concern for being infected with HIV or other STIs compared to pregnancy. This phenomenon, also reported by Miller et al. (24) and Daniel et al. (35) in Kenyan studies, manifests as a cause for concern particularly in this region where HIV rates are high, affecting 21% of adults in Siaya county (36). HIV continues to be the leading cause of mortality amongst female adolescents in SSA (37). Narratives from our participants and those of Miller et al. (24) indicate this phenomenon of comparison is relative to the visible nature of pregnancy which brings condemnation and shame, whilst living with HIV or other STI can likely go unnoticed without confirming sexual activity, important to the AGYW in our population. Participants also generally expressed confidence that treatment is available and effective for HIV and STIs, a testament to treatment scaleup (38). Nevertheless, in Kenya, AGYW continue to become infected with HIV at a disproportionate rate, have the lowest knowledge levels of prevention of mother to child transmission, lowest levels of Pre-Exposure Prophylaxis (PrEP) use, and highest levels of self-reported STI symptoms compared to older women (21).

Given the impoverished setting and the legal status of abortion in Kenya, while girls frequently spoke of their own or their peers seeking to terminate a pregnancy, barriers to accessing services with a skilled provider were primarily costs and fear of legal repercussions. Therapeutic abortion is legal to preserve life in Kenya, but women and girls are discouraged from seeking care, face harassment and even prosecution, while healthcare workers also face intimidation when providing such services (39). Contrary to other studies (40) there was no mention of abortion services being more difficult to access because of COVID-19 restrictions, and while we did not question this directly, it may stem from lack of demand of official services with reliance on clandestine social networks due to reasons stated above. Ultimately our participants reported that girls had little recourse but to access either illegal covert services from a clinician, at risk to both them and the provider, or to attempt an unsafe abortion without any medical supervision. Based on our participants’ perceptions, our results suggest a high number of girls may have been, and will continue to be, at risk from unsafe abortion practices until both legal and financial amendments are made to abortion service provision in Kenya.

Many studies abound with data regarding the psychosocial impact of abortion stigma (29, 41). However, our participants spoke more about the stigma of pregnancy. They spoke of pressure from parents, usually mothers, and boyfriends and sex partners, and their own specific reasons for not wanting to continue with the pregnancy. We were struck by the frequency and normalcy by which girls talked openly about having or attempting an abortion. This was unexpected as abortion is traditionally a hidden taboo and those seeking abortion risk discrimination and social exclusion, including from their own peers. Whether this represents a lasting or temporary reaction in these girls’ views on abortion, made more necessary or permissive by the pandemic and subsequent increases in pregnancies, there is need of continued and broader evaluation, pursuing perspectives from parents and healthcare providers. The long-term effects of increased numbers of girls experiencing abortions are, as yet unknown. Quantification using tools like the Demographic Health Survey would allow population level capture and representativeness, but respondents may be likely to underreport socially censored behaviour (42). It is likely mixed methods studies combined with indirect surveillance (e.g., abortion complications, confidante approach, list experiments) (43) will be most effective at gaining further insight into the scope and nature of abortion among AGYW in Kenya, and other places where it is criminalized, restricted, or highly stigmatized.

Some abortifacients that girls and men commonly spoke of would not result in the termination of a pregnancy (e.g., taking the antimalarial drug Coartem), and aligned with the frequency with which girls talked about failed abortions. Despite the abundance of literature on abortion, there is a paucity around failed abortion. The few studies undertaken (44, 45), suggest those who suffer one are more likely to display lack of maternal instinct and experience poor mental health consequences. Qualitative study by Oluseye et al. (46) in Nigeria where abortion is also illegal, found participants suffered from the stigma of being a young unmarried mother, as per the fears of our own participants. Unlike our participants they spoke about their experience of failed abortion, with emotion, stigma and distress being evident in their narratives. The difference in the narratives as compared with our participants may be due to our gathering of data through FGDs rather than individual in-depth interviews (IDIs) which may have facilitated deeper questioning, probing and privacy to unburden without a group of peers who may judge. The IDIs in the Oluseye et al. study (46) were done retrospectively involving a time span which may have given both a long-term perspective and maturity of participants. The non-emotional discussion among our participants may reflect the recency of experiences, or a current state of ‘normalcy’ post pandemic. Further research is key to understand the physical and psychological consequences of those who undergo a failed abortion, to prevent and mitigate short- and long- term adverse impacts to AGYW and their offspring.

Underlying these narratives our interviews with community men brought to light some contradictory views and behaviours. For example, among the few men who voiced using condoms, they noted needing them to prevent girls from giving *them* infections, without considering girls’ risk of contracting STIs and HIV. Sexual double standards have been described in the literature, identifying some sexual behaviours as acceptable for men, but not for women or girls who are then vilified (47)*.* Another example includes the sense of injustice some men expressed in being blamed for impregnating AGYW in the community while admitting to having sex with them. We note this was not all men and that these accounts were not challenged in the discussion groups. Within a male environment, participants may have felt compelled to display a more masculine or male hegemonic attitude than had we interviewed them individually. This may be a limitation of using FGDs to collect data and where men (and girls) may prefer to acquiesce with the dominant views. Equally this may stem from the patriarchal setting where our study was set, with men at ease with gendered attitudes being the norm (48). Using a female moderator may have influenced the discussion, either tempering stated views or possibly inciting them. It may therefore have been useful to undertake some discussions led by a male moderator to assess any gendered moderator effect. Another potential limitation include that participants were asked to recall phenomena from during the COVID19-related school closures, which ended approximately 15–18 months prior to these FGDs, and thus sentiments may have changed in retrospect.

## Conclusion

In this region of western Kenya girls will remain at high risk of pregnancy and unsafe abortion until community attitudes and knowledge are challenged. AGYW and men need to be provided with information and education to dispel myths and misinformation regarding contraception use and family planning methods, and address inequities in gender norms. Safe, legal and affordable abortion needs to be provided. Participant follow-up is required to assess long term physical and psychological consequences of the high number of pregnancies and abortions, particularly amongst those who had a failed abortion.

## Data Availability

The datasets presented in this study can be found in online repositories. The names of the repository/repositories and accession number(s) can be found below: the datasets supporting the conclusions of this article are available in the University of Illinois at Chicago repository, Dataset: Mehta, Supriya (2024). Cache2 Focus Group Discussion Transcripts AGYW and Community Males. doi: 10.25417/uic.26495884.v1.

## Footnotes

^1^Participants used the term ‘doctor’ to refer to any type of clinician who offers medical services
